# Supplementary material for: DISCERN: deep single-cell expression reconstruction for improved cell clustering and cell subtype and state detection
Source: Genome Biol. 2023 Sep 20;24:212. doi: 10.1186/s13059-023-03049-x (PMC10510283; doi:10.1186/s13059-023-03049-x)
Supplement: Supplementary file 2 — Additional file 2. Supplemental tables [24, 40, 42, 49, 89, 90, 93–96]. [file 13059_2023_3049_MOESM2_ESM.pdf]

DISCERN: deep single cell expression reconstruction for improved cell clustering and cell subtype and state detection.
# – Supplemental tables –

Fabian Hausmann,[1,2,*] Can Ergen-Behr,[1,2,4,*] Robin Khatri,[1,2]
Mohamed Marouf,[1] Sonja Hänzelmann,[1,2,3] Nicola Gagliani,[4,5,6,7]
Samuel Huber,[4,5] Pierre Machart,[1,2*] Stefan Bonn,[1,2,5*]

[*]These authors contributed equally to this work.
[1]Institute of Medical Systems Biology, University Medical Center Hamburg-Eppendorf,
Martinistraße 52, 20246 Hamburg, Germany
[2]Center for Biomedical AI,University Medical Center Hamburg-Eppendorf,
Martinistraße 52, 20246 Hamburg, Germany
[3]III. Department of Medicine,University Medical Center Hamburg-Eppendorf,
Martinistraße 52, 20246 Hamburg, Germany
[4]I. Department of Medicine,
University Medical Center Hamburg-Eppendorf,
Martinistraße 52, 20246 Hamburg, Germany
[5]Hamburg Center for Translational Immunology (HCTI),
I. Department of Medicine,University Medical Center Hamburg-Eppendorf,
Martinistraße 52, 20246 Hamburg, Germany
[6]Department of General, Visceral and Thoracic Surgery,
University Medical Center Hamburg-Eppendorf,
Martinistraße 52, 20246 Hamburg, Germany
[7]Section of Molecular Immunology und Gastroenterology,
I. Department of Medicine, University Medical Center Hamburg-Eppendorf,
Martinistraße 52, 20246 Hamburg, Germany

[*]To whom correspondence should be addressed; E-mail: sbonn@uke.de.

Table S1: *Overview of all single cell and bulk sequencing datasets used in this study.* The table shows the dataset name, size of the dataset, the sequencing technology, cell types as annotated in the original study and a hyperlink to the publication.

Dataset	*Method*	*Cell Types*	*Publication or Download link*
**pancreas** (8569 cells)	SMARTSeq2, Fluidigm C1, CelSeq, CelSeq2, inDrops	alpha, beta, ductal, acinar, delta, gamma, activated_stellate, endothelial, quiescent_stellate, macrophage, mast, epsilon, schwann	[89]
**difftec** (31 021 cells)	10x Chromium v2, 10x Chromium v3, SMARTSeq2, Seq-Well, inDrops, Drop-seq, CelSeq2	Cytotoxic T cell, CD4$^+$ T cell, CD14$^+$ monocyte, B cell, Natural killer cell, Megakaryocyte, CD16$^+$ monocyte, Dendritic cell, Plasmacytoid dendritic cell, Unassigned	[90]
**covid-lung** (56 645 cells)	10X Genomics Chromium Single Cell 5'v1.1	CD8 T, TREG, CD4_CD8 proliferating, B cell, CD4_TCM, TRM1, TR1, CD8_TCM, T senescent, CD8_TEM, TEM17, T antiviral, alveolar MΦ, TRM17, M1, CD4_CD8 stressed TCM, CD4_TSCM, MAIT, Innate like, Neutrophils, doublets, CD4_CD8 lnc rich, aged Neutrophils, M1 HSP$^+$, Mast, DC, M1 Mono-derived, M2 profibrotic, Epithelial, Neutrophil, Macrophage	[24]
**covid-blood** (83 709 cells)	10X Genomics Chromium Single Cell 5'v1.1	CD3$^+$ cells	[24]
**citeseq** (6592 cells)	10x Genomics Single Cell and CITE-seq	B cells, CD4 T cells, NK cells, CD14$^+$ Monocytes, FCGR3A$^+$ Monocytes, CD8 T cells	`https://www.ncbi.nlm.nih.gov/geo/query/acc.cgi?acc=GSE100866` `https://github.com/YosefLab/scVI-data/raw/master/pbmc_metadata.pickle`

Table S1: *Overview of all single cell and bulk sequencing datasets used in this study continued.*

Dataset	***Method***	***Cell Types***	***Publication or Download link***
**bulk** (9852 cells)	SMART-seq v4	Naive CD4, Memory CD4, TH1, TH2, TH17, Tfh, Fr. I nTreg, Fr. II eTreg, Fr. III T, Naive CD8, Memory CD8, CM CD8, EM CD8, TEMRA CD8, NK, Naive B, USM B, SM B, Plasmablast, DN B, CL Monocytes, Int Monocytes, NC Monocytes, mDC, pDC, Neutrophils, LDG	[40]
**covid-blood-severity** (636 836 cells)	10X Genomics Chromium Single Cell 5'v1.1	ASDC, B_exhausted, B_immature, B_malignant, B_naive, B_non-switched_memory, B_switched_memory, C1_CD16_mono, CD4.CM, CD4.EM, CD4.IL22, CD4.Naive, CD4.Prolif, CD4.Tfh, CD4.Th1, CD4.Th2', CD4.Th17, CD8.EM, CD8.Naive, CD8.Prolif, CD8.TE, CD14_mono, CD16_mono, CD83_CD14_mono, DC1, DC2, DC3, DC_prolif, HSC_CD38neg, HSC_CD38pos, HSC_MK, HSC_erythroid, HSC_myeloid, HSC_prolif, ILC1_3, ILC2, MAIT, Mono_prolif, NKT, NK_16hi, NK_56hi, NK_prolif, Plasma_cell_IgA, Plasma_cell_IgG, Plasma_cell_IgM, Plasmablast, Platelets, RBC, Treg, gdT, pDC	[49]

Table S2: *Detailed quality and batch information for all single cell and bulk sequencing datasets used in this study.* For each batch, the number of cells, the mean number of counts per cell, and the mean number of expressed genes per cell are listed. For the difftec dataset, the batch names were slightly adjusted. Their published batch names are written in brackets.

**Dataset**	**Batch**	**Number of cells**	**Mean number of counts per cell**	**Mean number of genes**
	smartseq2	2394	451021.4	6214.0
	fluidigmc1	638	1580155.4	8127.4
**pancreas**	celseq	2285	11161.1	3466.8
	celseq2	1004	23394.2	5274.9
	indrop	8569	5828.2	1887.2
	dropseq (pbmc1_Drop-seq)	3222	1282.0	676.0
	indrops (pbmc1_inDrops)	3222	566.3	362.4

difftec

Table S2: *Detailed quality and batch information for all single cell and bulk sequencing datasets used in this study continued.*

Dataset	Batch	Number of cells	Mean number of counts per cell	Mean number of genes
	seqwell (pmbc1_Seq-Well)	3222	1035.3	567.2
	chromium-v3 (pbmc1_10x Chromium (v3))	3222	4891.3	1514.1
	chromium-v2 (pbmc1_10x Chromium (v2) A)	3222	2120.0	795.4
	chromium-v2B (pmbc1_10x Chromium (v2) B)	3222	2512.4	870.8
	smartseq2 (pbmc1_Smart-seq2)	253	385914.3	2434.6
	celseq2 (pbmc1_CEL-Seq2)	253	6057.3	2585.4
	dropseq-2 (pbmc2_Drop-seq)	3362	2141.0	977.7
	seqwell-2 (pbmc2_Seq-Well)	551	692.6	421.8
	smartseq2-2 (pbmc2_Smart-seq2)	273	292924.3	2795.4
	celseq2-2 (pbmc2_CEL-Seq2)	273	5949.3	2556.6
	chromium-v2-2 (pbmc2_10x Chromium (v2))	3362	2860.7	1131.4
	indrops-2 (pbmc2_inDrops)	3362	1249.5	619.5
**covid-lung**	Bacterial	14591	9627.2	1617.4
	SARS-CoV-2	42054	10284.4	1719.5
**covid-blood**	Bacterial	22199	5861.6	1703.0
	SARS-CoV-2	61510	5388.6	1700.7
**citeseq**	citeseq	6592	1391.8	797.8
**bulk**	bulk	9852	881440.6	13103.8
	cambridge	130637	4798.9	1485.9
**covid-blood-severity**	ncl	431733	3520.0	1276.1
	sanger	74466	3640.2	1445.1

Table S3: Antibodies used in the CITE-seq experiments of the citeseq dataset (see table S1 & table S2).

Antibody	Clone	Supplier	Target Protein	Target Gene
CD3e	UCHT1	BioLegend, USA	CD3	*CD3E*, *CD3D*
CD19	HIB19	BioLegend, USA	CD19	*CD19*
CD4	RPA-T4	BioLegend, USA	CD4	*CD4*
CD8a	RPA-T8	BioLegend, USA	CD8	*CD8A*
CD56	MEM-188	BioLegend, USA	NCAM1	*NCAM1*
CD16	B73.1	BioLegend, USA	FCG3A	*FCGR3A*
CD11c	B-ly6	BD Pharmingen, USA	CD11c	*ITGAX*
CCR7	150603	RD Systems, USA	CCR7	*CCR7*
CCR5	J418F1	BioLegend, USA	CCR5	*CCR5*
CD34	581	BioLegend, USA	CD34	*CD34*
CD14	M5E2	BioLegend, USA	CD14	*CD14*
CD10	HI10a	BioLegend, USA	NEP	*MME, CD10*
CD45RA	HI100	BioLegend, USA	PTPRC, CD45RA	*PTPRC*
CD2	RPA-2.10	BioLegend, USA	CD2	*CD2*

CD57	H-NK1	BioLegend, USA	B3GA1, CD57	*B3GAT1*

Table S4: *Proportion of $CD4^+$ T helper cell subtypes (TH1, TH2, TH17, and Treg) identified in the reconstructed-hq data, bulk-hq training data, and published ground-truth cell fractions in blood.* The proportions are calculated with respect to the total number of PBMCs. Five studies were used to compare the proportions in the reconstructed data with existing literature. These studies estimate one or more of these subtypes using FACS and subsequent cell activation. Overall, the proportions of T helper cell subtypes in the reconstructed-hq data correlate very well with the published information. It is also noteworthy that the bulk-hq training data contains almost a 10-fold higher proportion of these T helper cell subtypes, proving once again that DISCERN reconstructs gene expression using inherent biological information in the cite-lq data. It does not seem to hallucinate cells that do not exist, or increase cell proportions based on the training data distribution.

**Source**	**TH1**	**TH2**	**TH17**	**Treg**
reconstructed-hq	0.007	0.006	0.015	0.024
bulk-hq	0.042	0.041	0.042	0.078
Vargas-Rojas *et al.* (2011) [93]	0.006 to 0.014	0.009 to 0.016	0.004 to 0.010	0.040 to 0.066
Sadeghi *et al.* (2020) [94]			0.033 ± 0.016	0.047 ± 0.021
Luo *et al.* (2017) [95]			0.015 ± 0.005	0.040 ± 0.010
Zhang *et al.* (2015) [96]	0.04 ± 0.029		0.018 ± 0.008	
Monaco *et al.* (2019) [42]	0.022 ± 0.007	0.011 ± 0.003	0.021 ± 0.007	0.020 ± 0.003

Table S5: *Disease-severity prediction performance using GBM classifiers trained on T cell fractions.* Column one (3 - classes) and column two (2 - classes) displays the classification performance using cell type fractions obtained with reconstructed covid-blood-severity-hq data for three classes (mild, moderate, and severe) and two classes (mild and severe), respectively. The third column (2 - classes published) shows the classification performance for the fractions based on the originally published T cell annotations. All classifications were conducted with a GBM using 25 runs of LOOCV (confidence intervals) and forward feature selection. The T cell subtypes used by the GBM for column one are CD8_EM, CD8_Tc2, TFH, TH17_cluster1, Treg_active. For column two the features are CD4_CM, CD4_cytotoxic, CD4_naive, CD8_EM, CD8_effector. For column three CD4.CM, CD4.Tfh, CD8.EM, NKT, and Treg cells were used. It is striking to observe the strong increase in performance in the 2 class case between reconstructed cell type (column 2) and originally published (column 3) cell type information.

	**3 - classes** (mild, moderate, severe)	**2 - classes** (mild, severe)	**2 - classes published** (mild, severe)	**2 - classes scANVI** (mild, severe)
**F1-Score (Micro)**	0.37 ± 0.01	$\mathbf{0.78 \pm 0.02}$	0.61 ± 0.01	0.64 ± 0.00
**F1-Score (Macro)**	0.36 ± 0.01	$\mathbf{0.75 \pm 0.03}$	0.58 ± 0.01	0.62 ± 0.00
**AUROC**	0.54 ± 0.00	$\mathbf{0.80 \pm 0.00}$	0.40 ± 0.01	0.63 ± 0.00
**Accuracy**	0.36 ± 0.01	$\mathbf{0.78 \pm 0.02}$	0.60 ± 0.01	0.64 ± 0.00

1
