# Supplementary material for: DISCERN: deep single-cell expression reconstruction for improved cell clustering and cell subtype and state detection
Source: Genome Biol. 2023 Sep 20;24:212. doi: 10.1186/s13059-023-03049-x (PMC10510283; doi:10.1186/s13059-023-03049-x)
Supplement: Supplementary file 3 — Additional file 3. Peer review history [1, 6, 8, 11–15, 34, 39, 41, 48, 51, 52, 57–60]. [file 13059_2023_3049_MOESM3_ESM.docx]

**Review history**

**First round of review**

**Reviewer 1**

DiSCERN implements a method for single cell RNA-seq in which single cell data in a low-quality dataset is imputed with the help of a related high-quality dataset. The method uses a deep learning, Wasserstein autoencoder, modified in several meaningful ways to adapt for scRNA-seq imputation. Wasserstein autoencoders have previously been used in image generation related applications. The motivation of the approach is that single cell data is often of low quality in terms of genes and UMIs detected per cell as well as the number of cells. The approach is meant to fill in missing data. Through multiple case studies DISCERN is shown to be effective in filling in missing data, often performing better than state of the art methods, and the approach could be useful in single cell analysis. However, there are several problems with the interpretation of results, gaps in benchmarking and areas in which the paper could be clarified.

1. One major difficulty in the application of this method could be in identifying a suitable high quality reference. The nature of the types of analysis conducted on the reconstructed data is likely to be important in determining the suitability of a reference data set, a reference data set that is good for one type of downstream analysis might not be suitable for another. For example, one type of analysis might be based on the quantification of different cell types in the low-quality sample. Another analysis might seek to compare the details of the gene expression programs between cells of the same type but derived from different donors. DISCERN might be more suitable for the first application than the second. It would be helpful to expand on this in some detail, showing the range of scenerios/reference data similarities in which DISCERN works well and where the performance starts to degrade. It might be useful to show how differential gene expression analysis (of genes in the same cell type under different conditions) much be influenced by the choice of the reference. It would also be useful to conduct a differential gene expression analysis between datasets reconstructed using the same low-quality dataset but different high quality references.

2. For Figure S6D the main text reads:
"Interestingly, DISCERN can not only recover biological expression information, but it is also able to apply sequencing method-specific effects after reconstruction. The smartseq2-hq dataset, for instance, displays nearly no ribosomal protein coding gene expression after sequencing as previously reported by [8], while data sequenced using InDrop, Cel-Seq, or Cel-Seq2 shows prominent ribosomal protein coding gene expression (Figure S6D, left panel). When reconstructing smartseq2-hq data to indrop-lq data, ribosomal protein coding gene expression is re-instantiated (Figure S6D, right panel)."
In an image style transfer task this effect might be desirable; in the biological research application it suggests a major problem with the method. It implies that the method might impute gene expression programs that are not in low quality data even if they are in the high quality data. The researcher would need to be able to discriminate the real imputed gene expression from the imagined. It could be that DISCERN is not suitable for identifying genes expression programs that might differ between the low-quality data and the reference. This problem needs to be further investigated and described.

3. Gene expression patterns can be shared between certain cell types, varying in degree of expression depending on genetics, the environment and other factors. For example, cell cycle or interferon response are common in many cell types. The reference data might have cells exhibiting a strong interferon response while the low-quality cells might have no interferon response. DISCERN might incorrectly reconstruct the interferon response in the low-quality data. How likely is this type of effect to occur, and what measures do investigators need to take to avoid misinterpreting artifacts introduced through the imputation method? Something like this is reflected in the text:

"To investigate the precision of gene expression reconstruction, we created an artificial dataset by dividing the smartseq2-hq pancreas data into two batches, smartseq-lq and smartseq2-hq. In the smartseq-lq batch, the top one KEGG pathways per cell type were removed by setting the expression of genes contained in these pathways to zero, while the smartseq2-hq remained unaltered. Therefore, a reconstruction of smartseq-lq data using smartseq2-hq reference (reconstructed-hq) should ideally recover the smartseq-lq expression to its original state, prior to the removal of the genes. DISCERN is able to reconstruct the mean expression for all cell types"
In this case the KEGG pathway was removed and recovered. In a real dataset how would one know if the KEGG pathway was recovered or just made up? This KEGG example points to a severe problem, not a desirable feature.

Section 2.2 of the manuscript: " It is important to highlight that the proper estimation of expression variation and the dropout rate is pivotal for the reliable computation of differentially expressed genes." suggests that differential gene expression analysis is a use case for DISCERN. The reconstruction of a gene signature that was deliberately removed from the low-quality data set and is falsely reconstructed seems to demonstrate that this is not a suitable use case for DISCERN.

4. There is also the possibility of the method being misused to generate data that supports a given hypothesis. For example, using a reference data set with features supporting the hypothesis it might be possible to transfer these features to the low-quality dataset. What are the controls needed to ensure that this is not being done? How should readers of papers based on imputed data interpret results?

5. In the Slingshot trajectory analysis the reconstruction accuracy of the trajectory isn't quantified. It would be better to use a benchmarking methodology such as Dynverse (https://github.com/dynverse) for a more systematic analysis. Section 2.2 of the manuscript describes an analysis in which a cell type is removed from the high quality data. It would be of interest to assess the effect of removing from the high quality data a cell type present in one branch of the differentiation trajectory, and trying to infer the trajectory based on the DISCERN reconstructed data. scVi - Palantir might be a suitable basis for comparison.

6. The analysis of the COVID-19 data is interesting and is potentially a good use case for DISCERN. However, the performance comparison is based on Seurat analysis, which might not be the state of the art. It would be of interest to compare the performance with classifiers built on scVi derived cell types. In addition, the single cohort leave-one-out cross validation scheme might be over-optimistic about classifier performance. As many single cell COVID-19 datasets are available in: Tian, Y., Carpp, L.N., Miller, H.E.R. et al. Single-cell immunology of SARS-CoV-2 infection. Nat Biotechnol 40, 30-41 (2022). https://doi.org/10.1038/s41587-021-01131-y
it would be better to carry out cross-cohort analysis, training the classifying on one cohort and testing on others, to demonstrate classification performance.

7. Many results are provided showing marker genes known to be expressed a given cell type and imputed in the correct cell type by DiSCERN. Although such results are reassuring, they don't provide a strong basis for systematically performance assessment. Therefore, it might be better to reduce the space in the paper dedicated to such results, perhaps using a summary table.

8. The abstract describes DISCERN in this way: "Here we present DISCERN, a novel deep generative network that reconstructs missing single cell gene expression using a reference dataset. DISCERN outperforms competing algorithms in expression inference resulting in greatly improved cell clustering, cell type and activity detection, and
insights into the cellular regulation of disease." This suggests that DISCERN simply reconstructs gene expression. Given the possibilities for inadvertent errors or intentional misuse, a more precise description should be provided and with a clearly stated warning of its limitations.

**Reviewer 2**

The authors present a new tool "Discern" which uses a reference dataset to reconstruct missing gene expression in (low quality) single-cell datasets. DISCERN is based on a generative neural network and can infer the expression of missing genes in a given dataset using a reference dataset.
Overall the manuscript is well written (in particular the discussion), the benchmarks are detailed and the accompanying supplementary figures are very thorough. The authors provide a number of test cases to show the generalizability of their approach. The software is documented and the user tutorials provided will be useful to a new user of the tool. I think the idea of the paper is intellectually interesting. As sequencing costs drop and we profile deeper and more cells, this tool can be useful in bridging the gap of missing discoveries in old shallow-sequenced datasets.

I have some major and minor comments that I would like the authors to address.

Major
---------


- The authors describe a "high quality (hq)" dataset as one having higher genes detected per cell (Page4 Line 37). But genes detected per cells is a function of the sequencing depth. Can the authors show the distribution of total sequencing depth of all the datasets. Relatedly, Smart-seq2 is not a UMI-based technology and as such it is not clear if achieving a high correlation when a non-UMI based dataset is projected onto a low sequencing depth UMI-based dataset is necessarily good. A better metric in my opinion is if the strength of differential expression of marker genes goes up when information is borrowed from the high-quality data or if there are more marker genes that can be discovered by this approach (on the lines of Figure S14).
- Related to last point and with respect to Figure 1 and associated supplementary figures, the authors should ideally use UMI-based datasets to perform any benchmarking.In their second benchmarking the authors use chromium-V3 as reference which in my opinion is a more sensible benchmarking. Instead of Figure 1 focusing on panacreas I think it makes more sense to focus on PBMC analysis in Figure 1 and move the pancreas analysis to supplementary figures - and motivate it by saying that such transfers are possible through DISCERN though it comes with the caveat of an over amplified signal (arising from use of a non-UMI reference dataset). In short, a fair comparison of whether or not signal to noise ratio goes up should be done using UMI-based datasets.
- For the PBMC analysis, the authors might consider including PBMCs profiled using Smart-seq3 data (PMID: 32518404).
- In Figure S6, only the average expression of different genes are shown. A complementary metric would be quantify this information at the single-cell level by plotting the percentage of cells of a particular cell type that express the given gene - this would highlight how DISCERN is influencing individual cells (the average might increase even if just one cell ends up getting most number of UMIs post DISCERN application). This information can also be plotted on UMAPs - highlighting what happens to expression of each of the gene on a per cell basis before an after correction (as is done in Figure S45)
- Section 2.3. The analysis of sn-lq to sc-hq makes little sense. These are measuring two different molecules (unspliced vs spliced assuming sc-hq quantified exons only). As such I am not sure the increased correlation of 0.97 is meaningful in any sense. it is not clear what the goal of this analysis is - do the authors anticipate users of snRNA-seq to use a scRNA-seq data to "improve" data quality? I think this section needs a bit of motivation and the authors should contexualize what the correlation means here given these are slightly different modalities (unspliced vs spliced)
- Section 2.3. The authors claim that some marker genes are hard to detect in kindney scRNA-seq data and this is likely due to dropout. Are these genes also lowly expressed? (this is determinable with the purified bulk RNA-seq) - the authors could highlight the expression of these genes using a KDE plot differentiating these from other marker that are detectible in scRNA-seq
- Section 2.4: I am not clear on why a GBM is required given the predictors are not many (5) (and have similar range) - did the authors try a linear approach and found it insufficient? Also what is the relative weight of the subclasses in predicting the severity status?

Minor
----------


- Page 5 Line 30: "When reconstructing smartseq2-hq data to indrop-lq data" is unclear. The authors switch between calling this approach reconstructing and projecting. I think the authors should add a statement in the beginning declaring what can be used interchangeably or choose one.
- Page 7 Line 28: "more than3" => more than three
- Page 9 Line 57 - reference for the bulk dataset is missing (though is present in Table S1) - it makes sense to include these inline too wherever appropriate
- The images in the pdf are very hard to read though I was able to download high resolution version from the Editorial manager
- Please consider making a website (using github pages) for the tool. This could simply be the tutorial vignettes hosted as html but would greatly increase the visibility and usability of the tool

**Authors’ response to reviewers**

We would like to thank the reviewers for their valuable and helpful suggestions. We are aware that this level of detailed reviewing takes considerable time and we greatly appreciate this. We firmly believe that the revised version of our manuscript “DISCERN - Deep Single Cell Expression ReconstructioN for improved cell clustering and cell subtype and state detection” is scientifically much stronger thanks to your efforts.

In the following point-by-point response we highlight all reviewer comments in black, author responses in blue, and manuscript changes in red font. We added one sentence before and after every addition in gray to give a bit more context.

We would also like to note that we updated all figures including a MAGIC DEG analysis to include imputed counts, which was not requested by the reviewers, All other figures involving MAGIC (e.g. mean expression, standard deviations, batch corrections, …) were not modified. We also added table 4 to the supplements, which was missing in the previous submission.

Reviewer #1:

We would like to thank the reviewer for the many very specific comments, suggestions, and criticisms. A reverberating point of the reviewer is how to ensure the correct choice of reference and how to (in) validate results obtained with DISCERN. Clearly, data integration and imputation, using any method, can generate artifacts, leading to the removal or invention of signals, which is a problem that requires thorough care.

While we show in a multitude of experiments that DISCERN is relatively robust to the choice of reference (e.g. in the newly added figures S6,S7,S27 and S28), we would like to highlight that we understand DISCERN as a means to generate hypotheses that require validation. We advocate several measures in the revised manuscript, e.g. validation using uncorrected data, reclustering using semi-supervised methods and confirmatory experiment. First, the choice of reference should be as close as possible to the biological setting of the lq data. Second, results of clustering, gene expression, and cell assignment should always be re-analyzed in the raw, uncorrected lq data. We highlight that findings from imputation can be leveraged to increase statistical power for tests on uncorrected data using semi-supervised integration with scANVI or clustering cells together for pseudobulk differential expression. Third, imputation results should be validated in additional experiments.

1. One major difficulty in the application of this method could be in identifying a suitable high quality reference. The nature of the types of analysis conducted on the reconstructed data is likely to be important in determining the suitability of a reference data set, a reference data

set that is good for one type of downstream analysis might not be suitable for another. For example, one type of analysis might be based on the quantification of different cell types in the low-quality sample. Another analysis might seek to compare the details of the gene expression programs between cells of the same type but derived from different donors. DISCERN might be more suitable for the first application than the second. It would be helpful to expand on this in some detail, showing the range of scenarios/reference data similarities in which DISCERN works well and where the performance starts to degrade.

Thank you for this valuable suggestion. Indeed it is important to highlight the scenarios in which DISCERN can be beneficial and we tried to achieve this by including many different experiments in the manuscript, ranging from total cell type overlap to no overlap, very different gene expression to similar. In more detail, the original manuscript included experiments with varying cell type overlaps or ratios of hq to lq batches (Figure 2C-E). In the revised manuscript, we have added an even more difficult example where we combined data sets from different organs (PBMC and Pancreas) where no cell type overlap was expected (Figure S27 & S28). DISCERN is able to keep the two organs apart and only a minor influence on cell type-specific marker genes can be observed. The batches coming from the Pancreas still get integrated. We added the following section to the manuscript, including the new figures S27 & S28:

Thus, DISCERN is able to keep existing expression correlations and improves the detection of cell type specific genes by reconstruction using an hq batch as reference.

Since DISCERN outperforms existing methods in the reconstruction with no cell type overlap (Figure 2F and Figure S26), we investigated the performance of DISCERN when there is no cell type overlap due to organ differences. When reconstructing the combined pancreas and pbmc_8k data set, DISCERN is able to keep the non-overlapping cell types separated and is thus not integrating pancreas and PBMC data together (Figure S27). However, the five pancreas batches are still integrated (Figure S27). To further investigate if the cell type-specific expression is kept intact, cell type-specific gene set enrichment was performed. Most of the expected enrichment scores are unchanged or slightly increased after reconstruction and only a few cell types show a decrease in the enrichment scores (Figure S28). Thus, DISCERN is very robust to batch-specific differences. However, we don’t recommend using DISCERN for data sets with very strong differences between batches, as cell type differences could get lost.

In conclusion, DISCERN is both a precise and robust method for expression reconstruction that outperforms existing methods by a significant margin.

Figure S27: tSNE representation of the pancreas and the pbmc_8k dataset for different reference batches. The pancreas dataset, including the five technology batches, was integrated with the peripheral blood mononuclear cell dataset pbmc_8k.

Figure S28: Difference in cell type-specific enrichment scores after reconstruction to single batches compared to uncorrected data. Gene set enrichment was calculated using PanglaoDB [1] on one-vs-rest cell type differential gene expression analysis. A visual representation of the underlying data is shown in fig. S27. The organ of origin is highlighted in blue (Pancreas) and orange (PBMC).

We hope that this additional experiment further lends credibility to DISCERN’s robust integration and imputation capabilities, providing yet another safeguard to the reader.

It might be useful to show how differential gene expression analysis (of genes in the same cell type under different conditions) might be influenced by the choice of the reference. It would also be useful to conduct a differential gene expression analysis between datasets reconstructed using the same low-quality dataset but different high quality references.

Thank you for these two constructive comments. We will address your suggestion to compare gene expression when the same cells are in different conditions in our response to

your third comment. In brief, we added a comparison of interferon-stimulated cells compared to controls after correction with DISCERN. We are able to maintain a positive correlation for interferon response genes, even if the reference data set was not stimulated. However, we would like to note that cases where biological differences and technical artifacts are strongly correlated are not well suited for batch correction and imputation algorithms, including DISCERN.

In this section we will focus on the use of different high-quality references. We added an experiment including all possible reconstructions for the indrop-lq batch of the pancreas data set. To assess the impact of these reconstructions we added an enrichment-based quantification of the cell type marker genes computed using differential gene expression analysis. Additionally, we were able to correlate this to the number of counts in each reference, which will be discussed in more detail in a later section of our responses. Our experiments provide strong evidence that DISCERN faithfully preserves marker genes and improves their detection when using various (suitable) high-quality reference data sets. We added the following to the manuscript, including the new figures S6 & S7:

The improved quality of indrop-lq data reconstructed to smartseq2-hq level is validated by the strong increase of genes expressed per cell, ranging from ≈2000 genes per cell in the uncorrected indrop-lq data to ≈6000 genes in the indrop-lq data after reconstruction (Figure S5).

To further evaluate the impact of the reference batch, we performed cell type marker gene detection and subsequent enrichment using cell type-specific gene sets (PanglaoDB [34]). We could see an increase in the expected cell type enrichment scores for nearly all reconstructions and cell types compared to uncorrected data (Figure S6). Furthermore, the median enrichment score is correlated to the total number of counts in the reference batch with the smartseq2 batch achieving the highest enrichment scores and indrop reference achieving the lowest enrichment scores (Figure S7). Thus we focused mainly on these two batches in the following analysis and benchmarks.

We next investigated the effect of reconstruction of three cell type-specific genes, before and after correction across the five pancreas datasets (Figures S8 and S9).

Figure S6: Normalized enrichment scores of the gene set enrichment results of cell type vs rest differential gene expression analysis in the indrop batch using different batches as reference. Enrichment was performed using pancreas-related cell type gene sets in PanglaoDB [1]. For each cell type, the expected cell type enrichment score is shown. The reference batch used in each of the comparisons is shown on the y-axis.

Figure S7: Median enrichment scores across cell types from fig. S6 in relation to the median number of counts per sequencing technology. The median number of counts per reference batch is shown on the x-axis and the median enrichment scores for all cell types, as displayed in fig. S6, using different batches as a reference shown on the y-axis.

2. For Figure S6D the main text reads: "Interestingly, DISCERN can not only recover biological expression information, but it is also able to apply sequencing method-specific effects after reconstruction. The smartseq2-hq dataset, for instance, displays nearly no ribosomal protein coding gene expression after sequencing as previously reported by [8], while data sequenced using InDrop, Cel-Seq, or Cel-Seq2 shows prominent ribosomal protein coding gene expression (Figure S6D, left panel). When reconstructing smartseq2-hq data to indrop-lq data, ribosomal protein coding gene expression is re-instantiated (Figure S6D, right panel)."

In an image style transfer task this effect might be desirable; in the biological research application it suggests a major problem with the method. It implies that the method might impute gene expression programs that are not in low quality data even if they are in the high

quality data. The researcher would need to be able to discriminate the real imputed gene expression from the imagined. It could be that DISCERN is not suitable for identifying genes expression programs that might differ between the low-quality data and the reference. This problem needs to be further investigated and described.

In general we completely agree with the reviewer, imputation as well as integration algorithms can potentially produce artifacts, which is why imputation and integration results should always be carefully inspected.

This is why we strongly advise to always investigate gene expression in the original data to validate imputation results, as we have performed in S33-35, S38 and S45-47. This will prohibit false conclusions due to imputation artifacts.

In addition, by and large DISCERN seems to be quite robust to changes in cell types or differences between organs (e.g. in 2D-F, S27 & S28, answer to comment 1) and can be used for hypothesis generation.

Please also see our initial comment to you regarding best practices of DISCERN use. We added the following section in the manuscript:

When datasets are large and complex, with many cell types in the lq and several non-overlapping cell types in the hq data, one should always ensure that training does not merge or mix non-overlapping cell types with other cells, by investigating that these cells keep their cell type-specific marker gene expression. While DISCERN is robust to differences between batches, we still recommend users to validate findings in the uncorrected expression data, as exemplified in Figs. S33 to S35, S38 and S45 to S47, and to perform experimental validation as recommended by [60]. Keeping these ‘checks and balances’ will usually result in good reconstruction results even for complex datasets.

One last brief comment on the specific experiment the reviewer relates to. While the smartseq2 dataset does not show ribosomal gene expression for purely technical reasons, the original cell does express ribosomal genes and a reconstruction of this expression is therefore a potentially desirable effect (similar to the experiments where specific genes of a pathway were artificially dropped out).

3. Gene expression patterns can be shared between certain cell types, varying in degree of expression depending on genetics, the environment and other factors. For example, cell cycle or interferon response are common in many cell types. The reference data might have cells exhibiting a strong interferon response while the low-quality cells might have no interferon response. DISCERN might incorrectly reconstruct the interferon response in the low-quality data. How likely is this type of effect to occur, and what measures do investigators need to take to avoid misinterpreting artifacts introduced through the imputation method?

While we can show that DISCERN is relatively robust to differences between lq and hq datasets, it is clear that over- and under-integration and artifact generation can occur at different levels. This is a general phenomenon in data integration and imputation. In the manuscript, we therefore strongly advise researchers to use DISCERN for hypothesis generation and highlight several safeguards. Please refer to our initial statement and the next response for further details.

Something like this is reflected in the text:

"To investigate the precision of gene expression reconstruction, we created an artificial dataset by dividing the smartseq2-hq pancreas data into two batches, smartseq-lq and smartseq2-hq. In the smartseq-lq batch, the top one KEGG pathways per cell type were removed by setting the expression of genes contained in these pathways to zero, while the smartseq2-hq remained unaltered. Therefore, a reconstruction of smartseq-lq data using smartseq2-hq reference (reconstructed-hq) should ideally recover the smartseq-lq expression to its original state, prior to the removal of the genes. DISCERN is able to reconstruct the mean expression for all cell types". In this case the KEGG pathway was removed and recovered. In a real dataset how would one know if the KEGG pathway was recovered or just made up? This KEGG example points to a severe problem, not a desirable feature.

Section 2.2 of the manuscript: " It is important to highlight that the proper estimation of expression variation and the dropout rate is pivotal for the reliable computation of differentially expressed genes." suggests that differential gene expression analysis is a use case for DISCERN. The reconstruction of a gene signature that was deliberately removed from the low-quality data set and is falsely reconstructed seems to demonstrate that this is not a suitable use case for DISCERN.

While we by and large agree that data integration and imputation has to be treated with care, we do have a slightly different notion regarding our gene and pathway removal experiments, which we deem very useful as they contain clear ground truth information. The reviewer comments that experiments in which a single KEGG pathway was removed and then reconstructed might point to a severe problem and not a desirable feature of DISCERN. In addition, the reviewer states that ‘the reconstruction of a gene signature that was deliberately removed from the low-quality data set and is falsely reconstructed seems to demonstrate that this is not a suitable use case for DISCERN’.

To answer this question we would first like to highlight the fundamental biological rationale for these experiments. In brief, the removal of a few pathway-determining genes still leaves the majority of cell-specific genes and pathways untouched. A good algorithm should therefore use the existing signature to robustly estimate the missing parts. Thus, DISCERN learns the conditional gene regulatory logic (e.g. if genes A, B, and C then gene D should be there as well).

Let us briefly exemplify this using the interferon experiment in our answer to comment 1 (see also novel Figs S6 & S7). Interferon expression might be absent but the presence of the upstream signaling cascade that induces interferon expression can inform DISCERN to correctly impute interferon expression (e.g. the presence of the necessary transcription factors).

Similarly, if we remove a single KEGG pathway, we do not remove upstream and downstream signals of this pathway, nor the strong interrelations to other cell determining pathways in the same cell. In other words, biological covarying genes are not removed by removing a KEGG pathway. DISCERN learns that those missing genes that are part of a bigger gene module in the specific cell are absent due to a technical effect (artificial removal).

Regarding the question of how one can be sure that the pathway should be there we would like to refer to our best practices stated in the revised document and in our first comment to you.

To clarify this we added the following section in the manuscript:

Therefore, a reconstruction of smartseq-lq data using smartseq2-hq reference (reconstructed-hq) should ideally recover the smartseq-lq expression to its original state, prior to the removal of the genes. We rationalized that these genes are important for cell identity and thus should be stably expressed in all cells of this cell type, disregarding the batch.

Thus, considering these genes to be expressed in a cell type-specific manner, DISCERN is able to reconstruct the mean expression for all cell types, achieving a correlation r = 0.99 (Figure 2A).

To address the point how DISCERN handles very strong differences in the reference batch, we performed another experiment using the pbmc_8k data set and another PBMC data set consisting of IFN-stimulated cells and non-stimulated cells (Kang et al., 2017 as provided by scGEN).

ISG15 in B cells	ISG20 in B cells	IFl6 in B cells	IFITM3 in B cells

**r t** 11 111 il 1_L1

ISG15 in CD14+ Monocytes	ISG20 in CD14+ Monocytes	IFl6 in CD14+ Monocytes	IFITM3 in CD14+ Monocytes

11I

*C* 4

0

:::: 3

*1* 2

l

0

-1

- 1 l1	J

11I	r11!

ISGlS in CD4 T cells	ISG20 in CD4 T cells	IFl6 in CD4 T cells	IFITM3 in CD4 T cells

*C* 4 111

0

·	3

*1*2

l

lH il 111 11 111

0

-1

ISGlS in CDS T cells	ISG20 in CDS T cells	IF16 in CDS T cells	IFITM3 in CDS T cells

^1^: ir111	j	**t** *l lll	ll* lli:f f :a

-1

'------r---------r--- '------r---------r---

1SGl 5 in Dendritic Cells	1SG20 in Dendritic Cells	IFl6 in Dendritic Cells	tFITM3 in Dendritic Cells

7

^1^: **t**t ll	r11 1r111	ll

-1

*'------T---*

ISG15 in FCGR3A+ Monocytes	ISG20 in FCGR3A+ Monocytes	IFl6 in FCGR3A+ Monocytes

^1^: t	1H ff111	r1il

-1

IFITM3 in FCGR3A+ Monocytes

\SGlS in NK cells	ISG20 in NK cells	IFl6 in NK cells	IFITM3 in NK cells

1il

**rt t**

)	*l* ***l*	re(B)O**

1

-1

Uncorrected	DISCERN	Uncorrected	DISCERN	Uncorrected	DISCERN	Uncorrected	DISCERN Reconstruction	Reconstruction	Reconstruction	Reconstruction

After reconstruction of the IFN data set (stimulated & control) with the pbmc_8k batch as a reference, we see a slightly increased IFN response also in some cells which express only a low amount of interferon in the uncorrected data. However, DISCERN is not correcting the interferon response in all cell types and several cells remain low in interferon response as expected. Furthermore, the gene expression of interferon-regulated genes remains higher in the stimulated cells, thus DISCERN is able to keep expression information that is specific to a batch to some extent. Therefore, a positive correlation of log2 fold changes between uncorrected and reconstructed expression of interferon-regulated genes (*ISG15*, *ISG20*, *IFI6*, *IFIT3*, *IFIT1*, *IFIT2*, *IRF7*, *MT2A*, *RSAD2*, *IFITM3*) is maintained. Thus we believe that DISCERN is robust across changes in cell types, batch ratios, and to some extent to biological differences in the batches. However, we would like to note that broad experimental influences like Interferon response in half of the IFN data set are harder to distinguish from sequencing technology-related differences. Thus, these cases are not well suited for batch correction and imputation algorithms, including DISCERN. We, therefore added the following section to our manuscript:

While DISCERN gave good reconstruction results using default parameters for most datasets we analyzed, we would like to highlight that the immense representational power of generative neural networks can remove or hallucinate biological information if not properly handled [6]. Especially, if there are biological differences between the high- and low-quality batches, these algorithms can falsely impute or remove these biological differences, since biological and technical differences cannot be distinguished. This is true for data integration [58, 59] as well as for expression reconstruction algorithms and we would highlight guiding principles for optimal results. For non-expert users, we would recommend the use of default settings and a careful selection of a related hq dataset, which should not show any huge biological differences. When datasets are large and complex, with many cell types in the lq and several non-overlapping cell types in the hq data, one should always ensure that training does not merge or mix non-overlapping cell types with other cells, by investigating that these cells keep their cell type-specific marker gene expression.

Here, validation using raw data and experimental validation is necessary, which we highlighted in our manuscript.

4. There is also the possibility of the method being misused to generate data that supports a given hypothesis. For example, using a reference data set with features supporting the hypothesis it might be possible to transfer these features to the low-quality dataset. What are the controls needed to ensure that this is not being done? How should readers of papers based on imputed data interpret results?

Thank you for raising this very important point. Altering data unethically to support one's notions of truth is obviously violating scientific ethics and should by all means be avoided. We would argue, however, that DISCERN relies on other data sets as reference, which at least to some extent represent biological ground truth. If we have the case, as you outlined, that the high-quality reference supports the hypothesis and the low-quality data set does not, the low-quality data set should not be used to verify the hypothesis. We have highlighted in the manuscript that users of imputation methods should always inspect the uncorrected data to substantiate imputed findings, as we have performed in S33-S35, S38, and S45-47, and by experimental procedures, as outlined in Luecken et al. (Molecular Systems Biology, 2019). Imputation methods can help with generating hypotheses but are not suited to validate hypotheses. It is easier as outlined above to detect lowly expressed genes after the definition of a potentially interesting group of cells based on the expression of a gene of interest. Imputation can help in identifying those cells without resorting to a less well-characterized set of genes (see e.g. RORC expression in PBMC example to identify TH17 cells). We recommend every reader of any imputation approach to check for these in a manuscript. We added a corresponding section in the main manuscript:

Keeping these ‘checks and balances’ will usually result in good reconstruction results even for complex datasets. We further advise every reader to validate imputation results by consulting the literature, as outlined in [60]. This will ensure reproducible results for every imputation method and prevents authors from drawing biased conclusions.

Following these principles, we were able to obtain novel insights into COVID-19 disease mechanisms and a new blood-based biomarker for disease severity we reconstructed two published datasets with DISCERN, Hamburg COVID-19 patients (covid-lung, -blood) and the COVID-19 cell atlas (covid-blood-severity).

5. In the Slingshot trajectory analysis the reconstruction accuracy of the trajectory isn't quantified. It would be better to use a benchmarking methodology such as Dynverse (<https://github.com/dynverse>) for a more systematic analysis. Section 2.2 of the manuscript describes an analysis in which a cell type is removed from the high quality data. It would be of interest to assess the effect of removing from the high quality data a cell type present in one branch of the differentiation trajectory, and trying to infer the trajectory based on the DISCERN reconstructed data. scVi - Palantir might be a suitable basis for comparison.

Indeed, the performed experiment using trajectory analysis are more qualitatively. We performed these experiments to show that continuous cell states, e.g.\ in CD4^+^ T cell differentiation, are preserved after DISCERN reconstruction using a reference (bulk RNA-seq) that does not contain those. It was not our aim to develop or improve trajectory

inference methods. Thus we reworded the section from Improving cell cluster, type, and trajectory identification to Improving cell cluster, type, and cell state identification and added the following changes and clarifications to the manuscript:

This lack of resolution in scRNA-seq impedes clustering, marker gene detection, and the identification of cell sub-types, a drawback that could be overcome using DISCERN’s expression reconstruction. We, therefore, compared CD4+ T cell (gene expression of CD4 > 1 and CD3E > 2.5) clustering and subtype identification using cite-lq and reconstructed-hq data.

Finally, we wanted to investigate if DISCERN could also enhance the detection of continuous cell states in the citeseq data. The differentiation of effector and other T helper cell subtypes involves several sub-states, which cannot be captured using bulk RNAseq. Thus, bulk RNAseq can only provide a limited reference for these cell types. We used trajectory analysis by Slingshot [48] to identify continuous differentiation pathways in the DISCERN reconstructed citeseq data and found five lineages that either pass through or terminate in the effector cell cluster in reconstructed-hq data (Figure 3C).

As another measure of the reliability of our cell states, we used Palantir, to compute terminal state probabilities for the same terminal clusters as in the slingshot bases trajectory analysis. We saw that the terminal state probability for most of these cell types was higher on average compared to uncorrected and Seurat reconstructed data. Especially TH17 and Active_TREG showed a strong increase in terminal probabilities, indicating that these trajectories can barely be found without DISCERN.

Furthermore, we removed CD4^+^ effector memory (CD4_EM) from the bulk RNA-seq data set, such that only the initial and terminal states are included in the high-quality reference.

Using this, we were able to recover similar trajectories, but with less pronounced differences between the cell subtype trajectories. Thus, also in the case of missing intermediate cell states, DISCERN is able to recover these states in the low-quality data, and trajectory methods are able to use this data to infer a corresponding trajectory.

6. The analysis of the COVID-19 data is interesting and is potentially a good use case for DISCERN. However, the performance comparison is based on Seurat analysis, which might not be the state of the art. It would be of interest to compare the performance with classifiers built on scVi derived cell types. In addition, the single cohort leave-one-out cross validation scheme might be over-optimistic about classifier performance. As many single cell COVID-19 datasets are available in: Tian, Y., Carpp, L.N., Miller, H.E.R. et al. Single-cell immunology of SARS-CoV-2 infection. Nat Biotechnol 40, 30-41 (2022). <https://doi.org/10.1038/s41587-021-01131-y>

it would be better to carry out cross-cohort analysis, training the classifying on one cohort and testing on others, to demonstrate classification performance.

We agree that the Seurat-based analysis might not be state of the art, thus we used scANVI and a kNN-based classifier to transfer our annotated labels and tested whether we were able to reliably detect similar cell types. The classifier after scVI embedding showed major agreement with our identified clusters. When applying scANVI as a semi-supervised method all cell types can be recovered except the Interferon signaling CD4 cells. We highlight that those cells are a true cell state highlighting three interferon-regulated genes. This emphasizes that scANVI can be used to validate identified cell types and for additional defined cell types careful validation of marker genes in raw expression space is essential. We thus added this statement about the robustness of your cell type identification in the manuscript:

Cells identified as TFH in the original publication show significant overlap with naive CD4 + T cells (defined on transcriptome and protein level) and CD4 + IL22 + cells (CD4.IL22) show marked overlap with TREG cells. To further assess the robustness of the cell type annotation we utilized scANVI [52] and a kNN-based classifier to compare with automated cell type annotations. Nearly all cell types could be detected using the automated approach (Fig. S57). These results confirm once more the precise and robust cell type identification that can be achieved with DISCERN.

Figure S57: Comparison of kNN-based cell type predictions on scANVI [39] transformed data to manually annotated cell types on the covid-blood-severity dataset.

One cell type where the recovery was not working, was Interferon-regulated cells (IFN_regulated). However, these cells show a high expression of Interferon response genes, which is not the case for other cell types. Thus, we think that in this case the annotation after DISCERN imputation was correct and these subtle differences are not well captured after scANVI integration.

We agree that a cross-cohort analysis involving multiple studies would be beneficial. However, ground truth annotation on the disease severity is challenging to get. Considering the supplemental table of Tian et al. (2022), for most papers different definitions of disease severity were used. Unifying these definitions would potentially be possible using patient records, which are not available for those studies. Thus, we decided not to include a different data set but split our covid-blood-severity data set by collection site. These data sets were processed at different hospitals, but the ground truth annotation on disease severity was already unified. Multi-center studies are generally seen as high-quality clinical trials to identify patient groups. We updated all the classification results for cross-collection site prediction and updated the manuscript as follows, including a new figure S59:

Training a GBM classifier on mild and severe cases substantially increased classification performance, reaching an AUROC of 0.80 and accuracy, and F1 score of 0.78 (Table S5, Figure 4F &G). Compared to the original T cell types and fractions reported (accuracy 0.61)

[48] or scANVI [51] predicted cell types (0.64), DISCERN reconstructed T cell fractions are 22 % to 25 % more accurate in the prediction of COVID-19 disease severity (Figure 4G, Table S5). DISCERN especially improved the performance when trained on the Ncl data set and predicted on the Cambridge data set (Fig. S59). This classification improvement is remarkable, given that DISCERN has no notion of disease severity when it reconstructs gene expression.

Figure S59: Disease-severity prediction using GBM classifiers trained on fractions of five T cell types of the covid-blood-severity-hq (left) data or published cell types (right). The cell types were selected using forward feature selection of the reconstructed covid-blood-severity-hq data. Confidence intervals (one standard deviation) were calculated using 25 runs of training and prediction. The disease category “critical” was combined with “severe” and “asymptomatic” with “mild”. Training was performed for each collection site individually and the prediction was performed on the other collection site.

We also updated Figure 4:

**F**: confusion matrix predicted on collection site Ncl with a classifier trained on Cambridge date and vise versa for disease severity prediction using GBM

classifiers trained on fractions of five T cell types (CD4 CM, CD4 cytotoxic, CD4 naive, CD8 EM, CD8 effector) using reconstructed covid-blood-severity-hq data.

7. Many results are provided showing marker genes known to be expressed in a given cell type and imputed in the correct cell type by DiSCERN. Although such results are reassuring, they don't provide a strong basis for systematically performance assessment. Therefore, it might be better to reduce the space in the paper dedicated to such results, perhaps using a summary table.

Thanks for this advice. We removed several detailed descriptions of marker genes from the main text and added those in the supplemental figures:

We first investigated the correspondence of gene expression prior (cite-lq) and post reconstruction (bulk-hq) with antibody-based surface protein labeling of (Figure 3A, Figure S33). For several proteins, the corresponding cite-lq gene expression was absent and cell type-specifically re-instantiated in the reconstructed-hq expression data with DISCERN (Figure 3A, Figure S33). In cases where cell type-specific gene and protein expression matched cite-lq data the expression in reconstructed-hq data was left unaltered (Figure S33). In general, we observed increased agreement between cell type-specific surface protein and gene expression after reconstruction, showing that DISCERN doesn’t invent or ‘hallucinate’ cell types but reconstructs the expected expression specific to each cell type.

We further corroborated these results by selecting eight known cell type-specific cytosolic proteins and investigated their expression before and after expression reconstruction (Figure S29). Several chemokine receptors showed the correct cell type-specific expression only after expression reconstruction (Figure S34) [41].

To further verify the cell type annotations, we extracted the top cluster-determining genes from the reconstructed-hq data. These genes showed high concordance with marker genes known from the literature (Figure S37). In addition, reconstructed CD4 naive, CD4 EM and CD4 CM show low expression of the genes important for the T helper subtypes TH1, TH2, TH17, Active TREG and IFN regulated. We further corroborated our cell type annotation of reconstructed-hq data by observing the expected expression of several established T cell subtype markers (Figure S38).

Further explanation for individual marker genes was added to the figure caption of S34: *MS4A1* is used as a marker for B cells, *IL7R* for CD4^+^ T cells), *MS4A7* for Monocytes, *GNLY* and *NKG7* for NK cells. These genes showed consistent expression before and after reconstruction. The chemokine receptors *CCR2*, a marker for Monocytes and activated T cells, *CXCR1*, a marker for NK cells, and *CXCR6* a marker for CD8^+^ T cells the correct cell type-specific expression after reconstruction, but low to no expression in the uncorrected data.

Further explanation for individual marker genes was moved from the main text to the figure caption of S37:

Members of the TNF-receptor superfamily are known to be expressed in T helper cell subtypes [11], which can be observed after reconstruction in TH17 cells and partially in TH1, TH2, Active TREG and IFN regulated cells. Similarly, reconstructed TH1 cells show the expected high expression of granzymes GZMK and GZMA [12], while MIAT and HLA expression are found in activated TREG cells after reconstruction (Active TREG cluster) [13, 14]. NOG expression is detected in reconstructed CD4 naive cells, as previously described [15].

However, we still believe that especially for the covid analysis section, the discussion of marker genes is very important to show the correctness of the cell type annotations. Thus, we left this part largely unchanged.

8. The abstract describes DISCERN in this way: "Here we present DISCERN, a novel deep generative network that reconstructs missing single cell gene expression using a reference dataset. DISCERN outperforms competing algorithms in expression inference resulting in greatly improved cell clustering, cell type and activity detection, and

insights into the cellular regulation of disease." This suggests that DISCERN simply reconstructs gene expression. Given the possibilities for inadvertent errors or intentional misuse, a more precise description should be provided and with a clearly stated warning of its limitations.

Thank you for this suggestion. We added a comment discussing the problem of biological differences in the abstract. However, due to limitations of the number of words, we hope to have addressed the points in more detail in the discussion.

Single cell sequencing provides detailed insights into biological processes including cell differentiation and identity. While providing deep cell-specific information, the method suffers from technical constraints, most notably a limited number of expressed genes per cell, which leads to suboptimal clustering and cell type identification. Here we present DISCERN, a novel deep generative network that precisely reconstructs missing single cell gene expression using a reference dataset. DISCERN outperforms competing algorithms in expression inference resulting in greatly improved cell clustering, cell type and activity detection, and insights into the cellular regulation of disease. We show that DISCERN is robust against differences between batches and is able to keep biological differences between batches, which is a common problem for imputation and batch correction algorithms. We used DISCERN to detect two unseen COVID-19-associated T cell types, cytotoxic CD4+ and CD8+ Tc2 T helper cells, with a potential role in adverse disease outcome.

We would like the reviewer to bear in mind that while potential artifact generation or the removal or introduction of signals is a general potential problem for integration and imputation algorithms, we cannot recall any other publication in which a concrete warning is issued in the abstract. We did our best to show that integration and imputation with DISCERN is at least on par with the best algorithms available to date and we hope that we addressed all concerns and remarks of the reviewer.

Reviewer #2: Major

- The authors describe a "high quality (hq)" dataset as one having higher genes detected per cell (Page4 Line 37). But genes detected per cells is a function of the sequencing depth. Can the authors show the distribution of total sequencing depth of all the datasets. Relatedly, Smart-seq2 is not a UMI-based technology and as such it is not clear if achieving a high correlation when a non-UMI based dataset is projected onto a low sequencing depth UMI-based dataset is necessarily good. A better metric in my opinion is if the strength of differential expression of marker genes goes up when information is borrowed from the high-quality data or if there are more marker genes that can be discovered by this approach (on the lines of Figure S14).

This is a great comment and we re-evaluated the marker gene detection in the pancreas data set in the newly added figures S6 & S7 accordingly.

In brief, we saw an increase in the enrichment score for cell type-specific markers when projecting the indrop batch using the smartseq2 batch as a reference. We used an unbiased estimation of marker genes coming from PanglaoDB, such that this is not influenced by the choice of reference or low-quality batch. We saw that especially smartseq2 and fluidigmc showed increased enrichment scores (S6) and that this is correlated to the median total number of counts per cell (S7). Interestingly using a UMI-based technology, like Cel-seq2, did not improve the detection of marker genes (measured by the enrichment score) as much as reconstruction using a non-UMI-based method such as smartseq2. We added the following part to the manuscript:

The improved quality of indrop-lq data reconstructed to smartseq2-hq level is validated by the strong increase of genes expressed per cell, ranging from ≈2000 genes per cell in the uncorrected indrop-lq data to ≈6000 genes in the indrop-lq data after reconstruction (Figure S5).

To further analyze the impact of the reference batch, we performed cell type marker gene detection and subsequent enrichment using a cell type-specific gene set (PanglaoDB [34]). We could see an increase in the expected cell type enrichment scores for nearly all reconstructions and cell types compared to uncorrected data (Figure S6). Furthermore, the median enrichment score is correlated to the total number of counts in the reference batch with the smartseq2 batch achieving the highest enrichment scores and indrop reference achieving the lowest enrichment scores (Figure S7). Thus we focused mainly on these two batches in the following analysis and benchmarks.

We next investigated the effect of reconstruction of three cell type-specific genes, before and after correction across the five pancreas datasets (Figures S8 and S9).

If the enrichment-based marker gene detection analysis (S6,S7) is performed without applying DISCERN for each batch individually, we can see that a similar tendency is already visible in the uncorrected data (figure below) further strengthening our choice of smartseq2 as the hq batch. Thus also smartseq2 provides better marker gene detection results in this dataset.

As requested we added the distribution of total counts (sequencing depth) per dataset below:

In line with our definition, the smartseq2 data set and the fluidigmc batch showed the highest number of detected counts in the pancreas data set. This is congruent with Wang et al. (2021) [<https://www.sciencedirect.com/science/article/pii/S1672022921000486>], showing that smartseq2 achieves a higher sensitivity using the same sequencing depth. We decided against using the fluidigmc batch, since it has a low number of cells (only 2 of 13 cell types have more than 50 cells) even if the marker gene detection is slightly improved in this data set. Unfortunately, downsampling-based experiments as conducted with the smartseq2 data (Fig. 2), are not possible with this low number of cells.

In the difftec dataset, the Cel-Seq2 and Smart-Seq2 batch showed a higher number of detected counts than Chromium (v3). The aim here was to apply DISCERN to various Chromium versions that are widely available. We wanted to highlight that using Chromium v3 as high-quality reference and v2 as a low-quality data can lead to improved marker gene detection.

- Related to the last point and with respect to Figure 1 and associated supplementary figures, the authors should ideally use UMI-based datasets to perform any benchmarking. In their second benchmarking the authors use chromium-V3 as reference which in my opinion is a more sensible benchmarking. Instead of Figure 1 focusing on panacreas I think it makes more sense to focus on PBMC analysis in Figure 1 and move the pancreas analysis to supplementary figures - and motivate it by saying that such transfers are possible through DISCERN though it comes with the caveat of an over amplified signal (arising from use of a non-UMI reference dataset). In short, a fair comparison of whether or not signal to noise ratio goes up should be done using UMI-based datasets.

While we do agree with the general notion that UMI-based datasets might further strengthen our analysis, we still believe that the smartseq2 batch is a suitable reference for the pancreas data set, as proven by the conducted marker gene detection experiments and literature surveys (see previous section). Since the pancreas dataset covers a wide range of sequencing technologies, has distinct cluster and includes technologies like indrop that are cost-wise preferable technologies but generally lack the resolution of more cost intensive approaches and therefore shows stronger batch dependency in (qualitatively seen in S2 and S10), we think that it is more suited for the main figure.

To investigate the marker gene expression in the difftec data set, including smartseq3 (see next response), we performed a similar experiment as for the pancreas data set using enrichment based on PanglaoDB. Except for bad performance on reconstructions using low-quality batches as a reference for CD4+ T cells, we found no big differences between the batches here. Smartseq2 and Chromium (v3) projections yield similar performance for marker gene detection.

We added the following paragraph to the manuscript to motivate and clarify our selection of a high quality reference:

We considered a dataset high quality if it showed a good tradeoff between the mean number of expressed genes and the cell number. For example, in the pancreas dataset the smartseq2 (6214.0 genes) and the fluidigmc1 (8127.4 genes) show the highest number of expressed genes, but the fluidigmc1 batch only consists of 638 cells compared to the smartseq2 batch with 2394 cells, thus we selected the smartseq2 batch as the high-quality batch. While this approach is working in most cases, also low-quality batches can have a high number of expressed genes, e.g. due to high levels of ambient RNA. In this case, the actual performance on downstream applications (e.g. marker gene detection as in S6 & S7) is more informative for assessing data quality but can only be performed after cell type annotation. However, DISCERN does not require the definition of a high-quality batch *a priori* and it can depend on the scientific question, e.g. a specific batch shows enriched expression of specific genes. In this case, the evaluation of multiple reconstructions with different “high-quality” batches can be useful and maybe require changing the high-quality batch after cell type annotation for best performance. Furthermore, the use of the dropout estimation procedure in the decoder allows to achieve a single-cell data-like distribution of the reconstructed data and thus is not as strongly violating statistical assumption of downstream analysis.

- For the PBMC analysis, the authors might consider including PBMCs profiled using Smart-seq3 data (PMID: 32518404).

As outlined in our prior response, we included Smart-seq3 data (PMID: 32518404) in our difftec data set. As you can see in the figures below, DISCERN was integrated the smartseq3 and difftec data and was able to achieve high correlation of mean expression when reconstructing using the smartseq3 data set.

However, we saw in the marker gene experiment (prior response) that reconstruction using this data set as a reference yields worse performance for CD4+ T cells than uncorrected data. When performing differential gene expression for marker gene detection of CD4 T cells (versus all other cells) in Smart-seq3, we found several ribosomal genes enriched, explaining why T cell-specific genes are not as enriched as in Chromium (v3) for example.

Thus, we think that this particular data set is not a good high-quality reference for our PBMC analysis and we would like to stick to our current data set which is used as a well-accepted reference data set for batch integration (i.e. used in the OpenProblems challenge https://openproblems.bio/). However, we think that in general Smart-Seq3 is a promising technology and should provide very good reference data sets for the use with DISCERN in future applications. We added the following section in the discussion to strengthen this point about the application of future sequencing technologies, e.g. Smartseq3:

A unique feature of DISCERN is the use of an hq reference to infer biologically meaningful gene expression. The quality and number of new sequencing technologies is increasing, e.g. Smart-seq3 [57]. DISCERN should enable the use of old, potentially low-quality, datasets together with new high-quality datasets and thus can improve the reusability of already existing datasets. This is especially useful for cases where biological material is limited, for example in rare diseases. While we consider this a main strength of DISCERN, the dependence on a suitable reference dataset might also limit its application. We took great care in this manuscript to mitigate this concern by showing how DISCERN is able to reconstruct gene expression for many different types of lq and hq pairs.

- In Figure S6, only the average expression of different genes are shown. A complementary metric would be quantify this information at the single-cell level by plotting the percentage of cells of a particular cell type that express the given gene - this would highlight how DISCERN is influencing individual cells (the average might increase even if just one cell ends up getting most number of UMIs post DISCERN application). This information can also be plotted on UMAPs - highlighting what happens to expression of each of the gene on a per cell basis before an after correction (as is done in Figure S45)

Thanks for this suggestion. We added a new figure S9 showing the tSNE representation of the genes in S8 ( previously S6). The tSNE representation shows the improved detection of marker genes (INS, REG1A, and SST) after reconstruction to the smartseq2 batch and the change in ribosomal protein abundance depending on the reference very well.

Figure S9: Gene expression of Insulin (INS), a ribosomal gene (RPS7), REG1A, and Somatostatin (SST) in the pancreas dataset. tSNE-based representation of data shown in fig. S8. The first column shows the uncorrected datasets, while the second and third column show reconstructions using DISCERN to the smartseq2 and the indrop dataset, respectively.

- Section 2.3. The analysis of sn-lq to sc-hq makes little sense. These are measuring two different molecules (unspliced vs spliced assuming sc-hq quantified exons only). As such I am not sure the increased correlation of 0.97 is meaningful in any sense. it is not clear what the goal of this analysis is - do the authors anticipate users of snRNA-seq to use a scRNA-seq data to "improve" data quality? I think this section needs a bit of motivation and

the authors should contexualize what the correlation means here given these are slightly different modalities (unspliced vs spliced)

- Section 2.3. The authors claim that some marker genes are hard to detect in kidney scRNA-seq data and this is likely due to dropout. Are these genes also lowly expressed? (this is determinable with the purified bulk RNA-seq) - the authors could highlight the expression of these genes using a KDE plot differentiating these from other marker that are detectable in scRNA-seq

This is a very relevant comment. Indeed, hnRNA and mRNA expression should be different and it is questionable if the experiment shown provides a good use case of DISCERN. We therefore removed these experiments from the manuscript.

- Section 2.4: I am not clear on why a GBM is required given the predictors are not many (5) (and have similar range) - did the authors try a linear approach and found it insufficient? Also what is the relative weight of the subclasses in predicting the severity status?

We included a linear (logistic regression) and our non-linear (GBM) classifier. We found that a linear classifier is not able to fully capture the differences in cell type proportions and their correlation with disease severity. Using LOOCV we achieved an accuracy of 0.76±0.00 compared to 0.82±0.01 for GBM. In response to the first reviewer, we changed the setting and made a cross-collection site comparison. Here we achieved an accuracy of 0.73±0.00 using the linear classifier and 0.78±0.02 using the GBM classifier. Using the new cross-collection site approach, we computed feature importance and found that for four of the five classes we found consistent importance scores. Especially CD8 effector memory T cells (CD8_EM) seem to be very important for the prediction of disease severity. These cells are for example known to be involved in long-term protection against COVID-19 [Adamo et al. (Nature, 2022)].

Minor

- Page 5 Line 30: "When reconstructing smartseq2-hq data to indrop-lq data" is unclear. The authors switch between calling this approach reconstructing and projecting. I think the authors should add a statement in the beginning declaring what can be used interchangeably or choose one.

We reformulated the sentence to “When reconstructing smartseq2-hq data with indrop-lq data as reference” and reformulated all “projecting” to reconstructing.

- Page 7 Line 28: "more than3" => more than three
- Page 9 Line 57 - reference for the bulk dataset is missing (though is present in Table S1) - it makes sense to include these inline too wherever appropriate

Thanks for the hints, this was corrected.

- The images in the pdf are very hard to read though I was able to download high resolution version from the Editorial manager

We are sorry to hear that the pdf figures were hard to read. We will do our best to include high-quality PDF versions in the main manuscript.

- Please consider making a website (using github pages) for the tool. This could simply be the tutorial vignettes hosted as html but would greatly increase the visibility and usability of the tool

Another great suggestion. We added a link to the read-the-docs and included HTML-rendered versions of the tutorial. To further increase the visibility and usability of DISCERN, it will be included in scvi-tools and the scverse ecosystem upon publication.

All original code has been deposited at github.com (https://github.com/imsb-uke/discern) and is publicly available as of the date of publication. Further documentation and usage examples can be found at (https://discern.readthedocs.io/). Any additional information required to reanalyze the data reported in this paper is available from the lead contact upon request.

**Second round of review**

**Reviewer 1**

The revised manuscript comprehensively addresses my comments.

One remaining issue concerns the practice of generating and testing hypotheses.
Using data twice or 'double dipping' can lead to overestimating significance, for some more details see: https://arxiv.org/abs/2207.00554 .
It would be worth briefly discussing this issue, and suggesting ways to mitigate the problem.

**Reviewer 2**

The authors have addressed all my comments. I have no additional suggestions.

**Authors’ response to reviewers**

Reviewer #1: The revised manuscript comprehensively addresses my comments. One remaining issue concerns the practice of generating and testing hypotheses. Using data twice or 'double dipping' can lead to overestimating significance, for some more details see: https://arxiv.org/abs/2207.00554 . It would be worth briefly discussing this issue, and suggesting ways to mitigate the problem. - Done
